# Study on the Molecular Mechanism of Interaction Between Perfluoroalkyl Acids and PPAR by Molecular Docking

**DOI:** 10.3390/toxics14010067

**Published:** 2026-01-11

**Authors:** Renli Wei, Huiping Xiao, Jie Fu, Yin Luo, Pengfei Wang

**Affiliations:** 1 Department of Environmental Engineering, Wenhua College, Wuhan 430074, China; 2 School of Environmental Science and Engineering, Huazhong University of Science and Technology, Wuhan 430074, China; jiefu@hust.edu.cn; 3 Chongqing Key Laboratory of Natural Product Synthesis and Drug Research, Innovative Drug Research Center, School of Pharmaceutical Sciences, Chongqing University, Chongqing 401331, China

**Keywords:** molecular docking, PFAA, PPARδ, correlation analysis, high-frequency amino acids, molecular dynamics simulation

## Abstract

Per- and polyfluoroalkyl substances (PFASs), as a class of “permanent chemicals” with high environmental persistence and bioaccumulation, have attracted much attention. In this study, we focused on the molecular mechanism of the interaction between perfluoroalkyl acids (PFAAs) and peroxisome proliferator-activated receptor δ (PPARδ). Using molecular docking, binding free energy calculation, and structural analysis, we systematically investigated the binding modes, key amino acid residues, and binding energies of 20 structurally diverse PFAAs with PPARδ. The results showed that the binding energies of PFAAs with PPARδ were significantly affected by the molecular weight, the number of hydrogen bond donors, and the melting point of PFAAs. PFAAs with smaller molecular weights and fewer hydrogen bond donors showed stronger binding affinity. The binding sites were concentrated in high-frequency amino acid residues such as TRP-256, ASN-269, and GLY-270, and the interaction forces were dominated by hydrogen and halogen bonds. PFAAs with branched structure of larger molecular weight (e.g., 3m-PFOA, binding energy of −2.92 kcal·mol^−1^; 3,3m_2_-PFOA, binding energy of −2.45 kcal·mol^−1^) had weaker binding energies than their straight-chain counterparts due to spatial site-blocking effect. In addition, validation group experiments further confirmed the regulation law of binding strength by physicochemical properties. In order to verify the binding stability of the key complexes predicted by molecular docking, and to investigate the dynamic behavior under the conditions of solvation and protein flexibility, molecular dynamics simulations were conducted on PFBA, PFOA, 3,3m_2_-PFOA, and PFHxA. The results confirmed the dynamic stability of the binding of the high-affinity ligands selected through docking to PPARδ. Moreover, the influence of molecular weight and branched structure on the binding strength was quantitatively verified from the perspectives of energy and RMSD trajectories. The present study revealed the molecular mechanism of PFAAs interfering with metabolic homeostasis through the PPARδ pathway, providing a theoretical basis for assessing its ecological and health risks.

## 1. Introduction

Per- and polyfluoroalkyl substances (PFASs) are a series of non-natural, artificially synthesized organic compounds that are widely used in various industries. However, due to their high thermal stability and chemical stability, PFASs can persist in the environment for a long time and are hardly biodegradable [1]. The common chemical characteristic of PFASs is their perfluoroalkyl part, which is stable in the environment so that PFASs are often referred to as “permanent chemicals” [2]. These substances have properties such as oil/water repellency and low surface tension, and are also widely used in industrial and consumer products, such as surfactants, lubricants, and food packaging materials. However, this persistence also leads to the long-term accumulation of PFASs in the environment, posing a potential threat to ecosystems and human health [3].

Perfluoroalkyl acids (PFAAs), as an important part of PFASs, possess unique chemical properties that make PFAAs both hydrophobic and oleophobic. PFAAs are widely used in various industrial and consumer products. Currently, the environmental pollution problems caused by PFAAs are becoming increasingly serious and have drawn significant attention [4,5,6]. Studies have found that PFAAs are difficult to be eliminated from the body through metabolism after entering the human body [7]. PFAAs’ toxic effects mainly include hepatotoxicity, endocrine disruption, neurotoxicity, immunotoxicity, carcinogenicity, lipotoxicity, etc. [8,9,10]. Wang et al. studied the toxic effects and mechanism of perfluoroether carboxylic acids (PFECA) [11]. The results showed that PFECA exposure would inhibit many cellular stress signals and cause liver enlargement, and perfluoro-3,5,7,9,11-pentaoxadodecanoic acid (PFO5DoDA)’s combined effect on glucocorticoid receptor GR and PPAR signals would lead to liver metabolic disorders. Although the hepatotoxicity of PFAAs has been identified, the related mechanism remains elusive. Lin et al. investigated the effects of PFASs on lipid metabolism in black-spotted frogs through a combined field and laboratory study [12]. The results showed that lipid accumulation induced by PFASs is alleviated by PPARα and LXRα antagonists.

Peroxisome proliferator-activated receptor (PPAR) is an important member of the nuclear receptor superfamily, mainly involved in regulating physiological processes such as energy metabolism, lipid homeostasis, and inflammatory responses [13]. PPAR plays a core role in maintaining metabolic homeostasis by integrating nutritional signals with gene expression and is an important target in metabolic research and environmental toxicology. Since PFASs have similar structures to endogenous fatty acids, PPAR may be an important target for PFASs to exert lipid toxicity. Chen et al. used molecular dynamics simulation technology and found that PFASs of different chain lengths could stabilize the ligand-binding domain conformation of PPARα through specific interactions, thereby affecting downstream gene expression [14]. Li et al. discovered that PFASs could directly bind to PPAR, increasing its in vitro transcriptional activity and binding ability, and transcriptional activity is closely related to the carbon chain length and functional group type of PFASs [15]. He et al. found that perfluorohexanesulfonic acid (PFHxS) would affect the dysregulation of various lipids and damage lipid homeostasis, leading to changes in the PPAR signaling pathway, and changes in downstream retinol, linoleic acid, and glycerophospholipid metabolism, verifying that PFAA would damage lipid homeostasis through PPAR16. These studies have revealed the structural basis of PFASs especially PFAAs interfering with the nuclear receptor signaling pathway at the molecular level, providing a mechanism explanation for understanding their lipotoxicity.

Molecular docking is an important method for the computer simulation of molecular interaction patterns, mainly used in the research of the binding model between small molecules and target proteins. This application can reveal the interaction mechanism between small molecule pollutants and degradation enzymes or other receptors from a microscopic perspective, as traditional experimental methods are almost unable to achieve such research. Recently, some researchers have introduced molecular docking into the field of environmental science and engineering to simulate the interaction between environmental pollutants and receptors [14,15,16]. For example, Wilson Maldonado-Rojas et al. introduced molecular docking into the study of the oxidation metabolism of persistent organic pollutants (POPs) and studied the interaction mechanism between bromodiphenyl ether and polychlorinated biphenyl environmental pollutants and human cytochrome P450 during the oxidation metabolism process [17]. Previous studies have utilized molecular docking to investigate the interaction between PFAS and PPAR subtypes (such as PPARα, β and γ), revealing some key binding residues and energy trends [2,18]. Most of the existing studies have focused on a few typical PFASs (such as PFOA, PFOS) or a specific subtype. There is still a relative lack of systematic combination patterns of structurally diverse PFAAs with PPARδ, especially in terms of quantitative analysis of the differences between branched and straight-chain isomers [15].

This paper systematically simulated the binding models between 20 different structures of PFAAs and PPARδ using molecular docking technology, and calculated the binding energies. On this basis, Spearman correlation analysis and Pearson correlation analysis were conducted, focusing on exploring the quantitative influence laws of the physical and chemical properties of PFAAs, such as molecular weight, number of hydrogen bond donors, melting point, and branched structure, on their binding affinity and mode. The key factors affecting the binding of PFAAs and PPARδ were analyzed, thereby revealing the essence of the interaction between PFAAs and PPARδ at the molecular level. In further research, the molecular dynamics (MD) method was used to verify the stability of the key complexes predicted by molecular docking, and the dynamic behaviors under solvation and protein flexibility were investigated. The research results will supplement the existing knowledge system and provide a more detailed molecular perspective for understanding the relationship between the structural diversity and toxicity differences in PFASs.

## 2. Materials and Methods

### 2.1. Molecular Docking

By reviewing the literature, it was determined to adopt the PPARδ protein receptor with PDB ID 3OZ0 [15], and the crystal structure of human PPARδ with a resolution of 3.0 Å (0.3 nm) was obtained from the Protein Data Bank (RCSB PDB, https://www.rcsb.org/) (accessed on 14 January 2025). The crystal structure of PPARδ was processed using PyMOL2.6 to remove water molecules and unwanted non-essential atoms [19]. Then, the AutoDock 4.2 was used to perform hydrogenation, charging, and filling in the amino acid residue gaps of the large molecule to improve the protein structure [20]. The docking methodology is based on a semi-empirical free energy force field (e.g., the MMFF94 force field for ligands) and utilizes a Lamarckian genetic algorithm (LGA) for conformational search. The scoring function approximates the binding free energy by considering intermolecular interactions such as van der Waals, hydrogen bonding, electrostatic, and desolvation effects [21]. Finally, the crystal structure of PPARδ was saved as the receptor structure for subsequent molecular docking processing.

20 PFAAs were selected as experimental group for molecular docking analysis, including straight-chain and branched carboxylic acids (pefluoroalkyl carboxylic acids) and sulfonic acids (perfluoroalkanesulfonic acids) [22]. The selection of these compounds was based on their chemical structural diversity and their widespread presence in the environment, aiming to comprehensively evaluate the interaction mechanism between PFASs and PPARδ. As shown in Table 1, the 20 PFAAs selected for molecular docking with PPARδ were divided into four groups: pefluoroalkyl carboxylic acids (linear) and pefluoroalkyl carboxylic acids (branched), perfluoroalkanesulfonic acids (linear), and perfluoroalkanesulfonic acids (branched).

**Figure 1:**
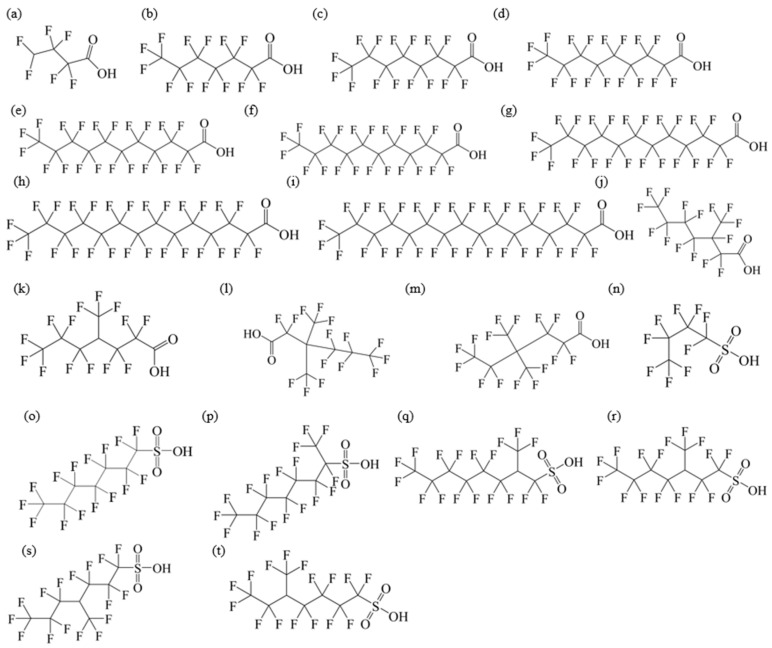
Chemical structures of PFAAs: (**a**) PFBA; (**b**) PFHxA; (**c**) PFHpA; (**d**) PFNA; (**e**) PFDA; (**f**) PFUnDA; (**g**) PFDoDA; (**h**) PFTeDA; (**i**) PFHxDA; (**j**) 3m-PFOA; (**k**) 4m-PFOA; (**l**) 3,3m_2_-PFOA; (**m**) 4,4m_2_-PFOA; (**n**) PFBS; (**o**) PFHxS; (**p**) 1m-PFOS; (**q**) 2m-PFOS; (**r**) 3m-PFOS; (**s**) 4m-PFOS; (**t**) 5m-PFOS.

The chemical structures and physicochemical property of PFAAs are summarized in Figure 1 and Table 2.

**Figure 2:**
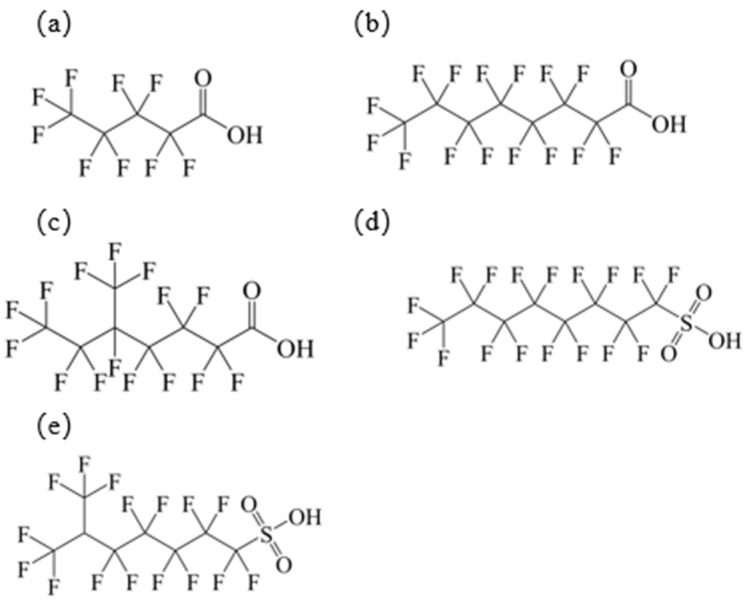
Chemical structures of PFAAs in validation group: (**a**) PFPA; (**b**) PFOA; (**c**) 5m-PFOA; (**d**) PFOS; (**e**) 6m-PFOS.

Five new small molecules (linear perfluoroalkyl carboxylic acids: PFPA, PFOA; branched perfluoroalkyl carboxylic acid: 5m-PFOA; linear perfluoroalkyl sulfonic acid: PFOS; branched perfluoroalkyl sulfonic acid: 6m-PFOS) were introduced as the verification group. Their physicochemical properties and structural formulas are shown in Table 3 and Figure 2.

Molecular docking was carried out as the following steps: downloaded 3D structure of PFAAs through the PubChem website (https://pubchem.ncbi.nlm.nih.gov/, accessed on 16 January 2025), then used AutoDock Tools to proceed to add all hydrogen atoms, set it as a ligand (automatically assign charges), detected torsion bonds and set torsion bonds, etc. Then exported it as a pdbqt file. Imported the processed protein crystal structure and the processed structure of PFAAs into AutoDock Tools, set the receptor and ligand, respectively, and adjusted the docking box (Grid Box) to ensure that the protein structure was fully within the docking box and the ligand was outside the docking box. Saved the set parameters and ran Autogrid. After Autogrid runs, set the docking parameters in the dock section. Then ran Autodock. After Autocok runs, a dlg file would be generated. In the file, it could be seen that whether the docking was successful, the lowest binding energy and the coordinates of the lowest binding energy site, etc. Opened the dlg file in ADT, selected the docking result with the lowest binding energy, exported it to the visualization software PyMOL for analyzing hydrogen bonds, key residues of interaction, bond lengths, etc., and finally visualized and exported the results.

### 2.2. Molecular Dynamics Simulation

The MD simulation was carried out according to the method described previously [23]. The GROMACS 2024.5 package was used to perform this simulation. The protein was parameterized with the AMBER99SB-ILDN force field, while ligand parameters were derived using the AMBER tools suite. The prepared complex was solvated in a cubic water box with a 1.0 nm margin, filled with SPC water molecules under periodic boundary conditions. System charge was neutralized by adding Na^+^ and Cl^−^ ions. Long-range electrostatic interactions were treated using the Particle Mesh Ewald method. An energy minimization step was first conducted to relieve any steric clashes or inappropriate atomic contacts. Subsequently, the system was gradually equilibrated through 100 ps of NVT (constant number, volume, and temperature) ensemble simulation followed by 100 ps of NPT (constant number, pressure, and temperature) ensemble simulation. Finally, a production MD simulation was carried out for 100 ns with a time step of 2 fs, maintaining constant temperature (300 K) and pressure (1 atm) throughout.

### 2.3. Statistical Analysis

Analysis of the experimented values were performed using Graphpad Prism 9.5 software (Graphpad, San Diego, CA, USA). The Pearson correlation and Spearman correlation were used to analyze the correlations between the interaction parameters between PFAAs and PPARδ and the physicochemical properties of PFAAs.

## 3. Results

### 3.1. Molecular Interactions of PFAAs in the Experimental Group with PPARδ

To explore the interaction between PFAAs and PPARδ, molecular docking was used to study their binding process. The binding energies and docking scores of all PFAAs with PPARδ are summarized in SI Table S1. The docking results showed that all PFAAs entered the binding pocket of PPARδ and bound at the same site, thus sharing many common interaction amino acid residues. The main forces between PFAAs and PPARδ were hydrogen bonds and halogen bonds. Most PFAAs could form hydrogen bonds with TRP-256, ASN-269, and GLY-270 in the ligand-binding domain of PPARδ.

**Figure 3:**
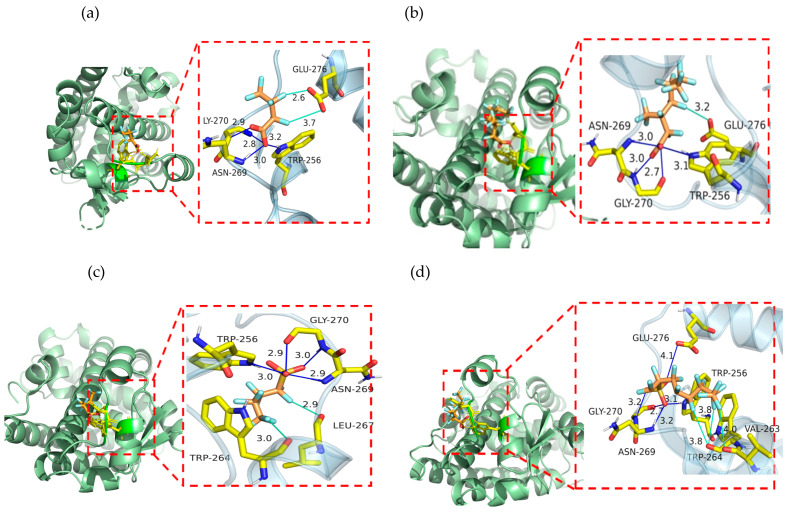
The docking interaction results of representative PFAAs with PPARδ: (**a**) PFBA; (**b**) 3m-PFOA; (**c**) PFBS; (**d**) 1m-PFOS. (For PFAAs: red-oxygen, orange-carbon, light blue-fluorine; for amino acid residue: yellow-carbon, blue-nitrogen, red-oxygen).

The docking results and binding data of representative PFAAs with PPARδ are shown in Figure 3 and Table 4. For PFBA (Figure 3a), the representative of perfluoroalkyl carboxylic acids (straight chain), the key amino acid residues involved in the interaction between PFBA and PPARα were TRP-256, ASN-269, GLY-270, and GLU-276, with the binding energy being −2.99 kcal·mol^−1^.

From Table 4 and Figure 3b, it is shown that 3m-PFOA, the representative of branched perfluoroalkyl carboxylic acids, formed a hydrogen bond and halogen bond with PPARδ, with the lowest binding energy being −2.92 kcal·mol^−1^. The key amino acid residues involved in the interaction between 3m-PFOA and PPARδ were TRP-256, ASN-269, GLY-270, and GLU-276.

From Table 4 and Figure 3c, it is shown that PFBS, the representative of perfluoroalkyl sulfonic acid (straight chain), formed a hydrogen bond and halogen bond with PPARδ, with the lowest binding energy being −4.78 kcal·mol^−1^. The key amino acids for the interaction between PFBS and PPARδ were TRP-256, TRP-264, LEU-267, ASN-269, and GLY-270.

From Table 4 and Figure 3d, it is shown that 1m-PFOS, the representative of perfluoroalkyl sulfonic acid (branched), formed a hydrogen bond and halogen bond with PPARδ, with the lowest binding energy being −3.65 kcal·mol^−1^. The key amino acid residues involved in the interaction between 1m-PFOS and PPARδ were TRP-256, VAL-263, TRP-264, ASN-269, GLY-270, and GLU-276, with the lowest binding energy being −3.65 kcal·mol^−1^.

The binding modes of other small molecules of PFAAs with different structures to PPARδ are presented in the Supporting Information (SI Figures S1–S16 and SI Tables S2–S17).

### 3.2. Structure-Activity Analysis of PFAAs in the Experimental Group with PPARδ

The interaction between PFAAs and PPARδ is shown in Figure 3. From the data mentioned above, it was obvious that, for different types of PFAAs, the binding energy varied a lot. Generally, the binding energy of perfluoroalkyl carboxylic acids (straight chain) (average value = −2.40 kcal·mol^−1^) was relatively higher that of other types of PFAAs (average value of branched perfluoroalkyl carboxylic acids = −2.90 kcal·mol^−1^, average value of perfluoroalkyl sulfonic acid (straight chain) = −4.20 kcal·mol^−1^, average value of perfluoroalkyl sulfonic acid (branched) = −3.81 kcal·mol^−1^). Based on the binding energy, the order of interaction strength between PFAAs and PPARδ was perfluoroalkyl sulfonic acid (straight chain) > perfluoroalkyl sulfonic acid (branched) > branched perfluoroalkyl carboxylic acids > perfluoroalkyl carboxylic acids (straight chain). Generally, the PFAAs with the group of sulfonic acid exhibited stronger binding affinity. From this result, it was clear that the key factor influencing the interaction between PFAAs and PPARδ might be the type of PFAAs.

**Figure 4:**
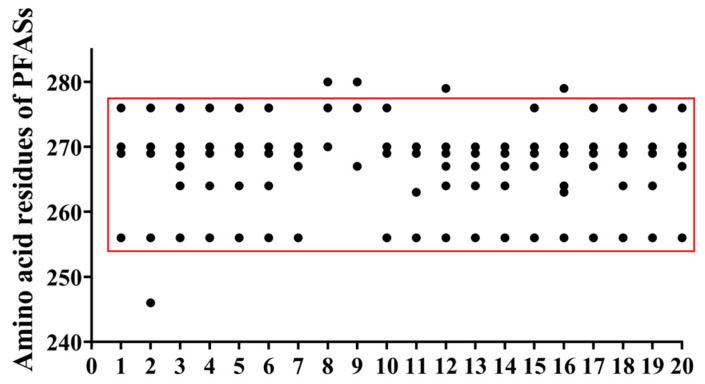
High-frequency amino acid distribution map: 1. PFBA; 2. PFHxA; 3. PFHpA; 4. PFNA; 5. PFDA; 6. PFUnDA; 7. PFDoDA; 8. PFTeDA; 9. PFHxDA; 10. 3m-PFOA; 11. 4m-PFOA; 12. 3,3m2-PFOA; 13. 4,4m2-PFOA; 14. PFBS; 15. PFHxS; 16. 1m-PFOS; 17. 2m-PFOS; 18. 3m-PFOS; 19. 4m-PFOS; 20. 5m-PFOS. (Black dot: amino acid residues; red frame: distribution region of high-frequency amino acid residues).

The statistics of amino acid residues involved in the interaction between PFAAs and the PPARδ are shown in SI Table S18. The distribution map of high-frequency amino acids is summarized in Figure 4. From this result, it was clear that the amino acid residues involved in the interaction between PFAAs and PPARδ were mainly distributed between TRP-256 and HIS-280. Among them, there were six frequently occurring amino acids, namely, TRP-256, TRP-264, LEU-267, ASN-269, GLY-270, and GLU-276. Most of the PFAAs except PFTeDA and PFHxDA could form hydrogen bonds with TRP-256, ASN-269, and GLY-270.

These six amino acid residues had completely different binding sites, interaction types, and corresponding bond lengths with each ligand. This might be due to the differences in the physicochemical properties of each ligand (mainly molecular weight, number of hydrogen bond donors, and melting point). For example, PFAAs (e.g., PFTeDA and PFHxDA) with higher molecular weight tended not to form hydrogen bonds with TRP-256 and GLY-270 due to steric hindrance effects, while smaller molecules (e.g., PFBA) were more likely to form strong halogen bonds with GLU-276.

### 3.3. Key Factors Influencing the Interaction Between PFAAs and PPARδ

The different molecular structures of PFAAs resulted in slight variations in their physicochemical properties (molecular weight, melting point, boiling point, density, flash point, topological polar surface area, number of hydrogen bond donors, and number of hydrogen bond receptors, as shown in Table 2). To clarify the relationship between the interaction strength of each PFAA with PPARδ and the physicochemical properties of PFAAs, Pearson correlation analysis and Spearman correlation analysis were conducted in Graphpad Prism 9.5 software on the physicochemical properties (molecular weight, melting point, boiling point, density, flash point, topological polar surface area, number of hydrogen bond donors, and number of hydrogen bond receptors) and interaction strength [24]. The correlation analysis result is summarized in Table 5. From this result, it was obvious that, according to the result of Pearson correlation analysis, the absolute value of binding energy was significantly positively correlated with molecular weight, the number of hydrogen bond receptors, melting point, and boiling point, and significantly negatively correlated with topological polar surface area. There was no correlation between binding energy and the number of hydrogen bond donors, density, or flash point.

**Figure 5:**
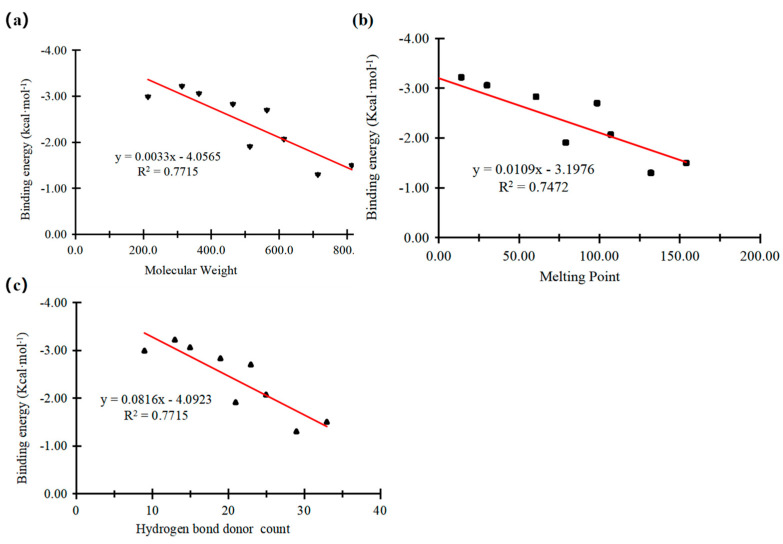
Correlation between binding energy of PFCAs with PPARδ and physicochemical properties of PFCAs: (**a**) molecular weight; (**b**) melting point; (**c**) number of hydrogen bond donors.

According to the calculation results of Spearman correlation analysis in Table 5, it could be seen that the absolute value of the binding energy was significantly positively correlated with molecular weight, the number of hydrogen bond donors, melting point, and flash point, and significantly negatively correlated with topological polar surface area. There was no correlation between binding energy and the number of hydrogen bond donors, density, and boiling point. Combined with Pearson correlation analysis and Spearman correlation analysis, the interaction strength between PFAAs with PPARδ might depend mainly on the combined effects of molecular weight, the number of hydrogen bond donors, melting point, and topological polar surface area. Further, a linear correlation analysis was conducted on the molecular weight, number of hydrogen bond donors, melting point, and binding energy of perfluoroalkyl carboxylic acids (straight chain) (PFCAs). The result is shown in Figure 5.

The influence of molecular weight of PFCAs on their interaction binding energy with PPARδ is shown in Figure 5a. The increase in molecular weight was accompanied by a decrease in the absolute value of binding energy, with R^2^ = 0.7715. This result indicated that the larger molecular weight of the pollutant was accompanied with a weaker interaction between PFCAs and PPARδ. The PFCAs with larger molecular weight exhibited weaker binding energy, possibly due to their spatial structure. The spatial restraints of PFCAs with larger molecular weight inhibited them from combining with PPARδ, while pollutants with smaller molecular weight might bind to PPARδ better due to their functional groups [15,25].

The influence of the melting point of PFCAs on their binding energy with PPARδ is shown in Figure 5b. A higher melting point led to a higher binding energy, with R^2^ = 0.7472, indicating that the interaction between the PFCAs and PPARδ becomes weaker as the melting point increased. The effect of the number of hydrogen bond donors of PFCAs on the binding energy with PPARδ is illustrated in Figure 5c. A greater number of hydrogen bond donors resulted in a smaller absolute value of the binding energy, with R^2^ = 0.7715. This result suggested that, as the number of hydrogen bond donors and the melting point increased, the interaction strength between PFCAs and PPARδ gradually weakened, which might reduce its adverse effects on the environment through the PPARδ pathway.

### 3.4. Molecular Interactions of PFAAs in the Validation Group with PPARδ

To verify the above correlation analysis results, five new PFAAs were introduced, including PFPA, PFOA in perfluoroalkyl carboxylic acids (linear), 5m-PFOA in perfluoroalkyl carboxylic acids (branched), PFOS in perfluoroalkyl sulfonic acids (linear), and 6m-PFOS in perfluoroalkyl sulfonic acids (branched). Their physicochemical properties and structural formulas are shown in SI Table S19.

Molecular docking results showed that the binding energy of PFPA, PFOA, 5m-PFOA, PFOS, and 6m-PFOS with PPARδ were −3.07, −3.89, −3.83, −3.53, and −3.56 kcal·mol^−1^. The binding models are shown in SI Tables S20–S24. The amino acid residues involved in the interaction between the validation group and the PPARδ were mainly distributed between LEU211 and GLU276. Among them, there were five frequently occurring amino acids, namely, TRP-256, TRP-264, LEU-267, ASN-269, and GLY-270 (SI Figure S17). The five frequently occurring amino acids in the validation group were all highly repetitive with the frequently occurring amino acids in the experimental group (TRP-256, TRP-264, LEU-267, ASN-269, GLY-270, GLU-276), with a repetition rate of 83.33%. These frequently occurring amino acids might be the key sites for the interaction between the PFAAs and PPARδ. Particularly, TRP-256 and GLY-270 had significantly enhanced hydrogen bond interactions, thereby significantly increasing the binding affinity of PFAAs.

From Table 6, it is shown that the intensity of the interaction between the PFAAs in the verification group and PPARδ decreased with the increase in molecular weight and the number of hydrogen bond donors, which was consistent with the conclusion obtained from the experimental group. This might be because the larger molecular weight led to a stronger steric hindrance effect, thereby weakening the binding ability of PFAAs to PPARδ. In addition, the increase in the number of hydrogen bond donors significantly increased the binding energy, indicating that fewer hydrogen bond donors could form a stronger hydrogen bond network with the key amino acid residues on PPARδ, thereby enhancing the binding affinity. This result was consistent with the molecular dynamics simulation results proposed by Chen et al., which reported that PFAAs formed specific interactions with the key amino acid residues of PPARδ through their terminal functional groups, thereby affecting the binding strength [26].

Due to the absence (N/A) of melting point data for PFAAs in the verification group, it was impossible to directly verify the conclusion of PFAAs in the experimental group, that “the larger the melting point, the weaker the interaction between PFAAs and PPARδ”. Future research could address this deficiency by introducing more physical and chemical property parameters (such as solubility, polarity, etc.).

### 3.5. MD Simulation

To verify the binding stability of the key complexes predicted by molecular docking and to investigate the dynamic behavior under solvation and protein flexibility, we conducted MD simulations on PFBA, PFOA, 3,3m_2_-PFOA, and PFHxA. Table 7 lists the molecular weights, molecular weight, presence of branched structures, and the fluctuation range of root-mean-square deviation (RMSD) for the four small molecules. The maximum root-mean-square deviation (RMSD) values for all systems were all less than 1, indicating that the docking conformations of all simulated systems were stable in the molecular dynamics simulation.

**Figure 6:**
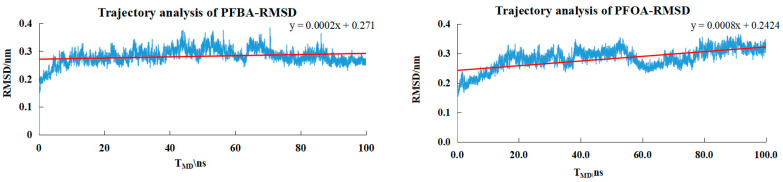

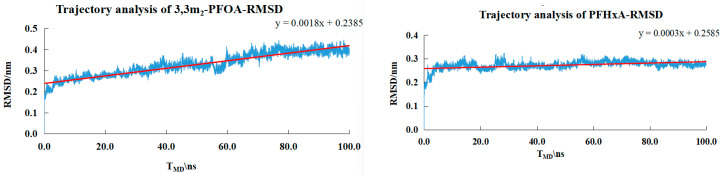
The trajectory RMSD values for the PFBA−PPARδ, PFOA-PPARδ, 3,3m_2_-PFOA-PPARδ, PFHxA-PPARδ systems.

As shown in Figure 6, the small molecules entered a plateau around 20 ns, and the RMSD fluctuated within the range of 0.28–0.45 nm. This indicated that the system had reached equilibrium at 20 ns, and the RMSD value remained relatively stable throughout the simulation process, without a continuous upward or downward trend, suggesting that the protein structure maintains a relatively stable conformation during the simulation.

By comparing the MD simulation results of PFBA, PFOA, and PFHxA, it could be observed that the molecular weights of PFBA, PFOA, and PFHxA were increasing. Although the conformations in the MD simulation were stable, the lowest binding energy showed a downward trend. This further confirmed that larger molecular weights of ligands usually implied greater steric hindrance effects, thereby restricting the binding of small molecules to the receptor.

For PFOA and 3,3m_2_-PFOA, which were compared under the premise of the same molecular formula by selecting small molecules containing branched structures, it could be seen that, although the RMSD value of 3,3m_2_-PFOA indicated that the conformation of the simulated system was stable, its fluctuation range was slightly larger than that of PFOA. This might be due to the presence of the branched structure, which led to a more compact and rigid spatial structure and also resulted in differences in binding energy for small molecules with branched structures (when performing molecular docking, the binding effect of small molecules with branched structures was not as excellent as that of small molecules with straight chains).

MD simulation not only verified the dynamic stability of the high-affinity ligands selected through docking and their binding to PPARδ, but also quantitatively confirmed the laws of the influence of molecular weight and branched structure on binding strength from the aspects of energy and RMSD trajectories, making up for the shortcomings of static docking. The simulated trajectories of this study have provided a stable foundation for further calculations involving free energy (such as MM-PBSA/GBSA). This analysis will be able to offer quantitative differences in free energy of binding, serving as a valuable complement to the current analyses of kinetic stability and interaction.

## 4. Conclusions and Prospects

In this study, the interaction between PFAAs and PPARδ was investigated by molecular docking. The results showed that all PFAAs could bind to PPARδ with reasonable binding energy and binding mode. The binding affinity of different types of PFAAs varied significantly. Generally, PFSAs exhibited better binding affinity (with average binding energy of −3.97 kcal/mol) with PPARδ than other types of PFAAs. Within the same group of molecules, like PFCAs, the binding energy was related to molecular weight and hydrogen bond donor count.

When PFAAs interacted with PPARδ, the interaction intensity was influenced by the physicochemical properties of PFAAs, especially the molecular weight, the number of hydrogen bond donors, and the melting point. This might be because a larger molecular weight usually implied a greater steric hindrance effect, thereby limiting the binding of PFAAs to PPARδ, while a greater number of hydrogen bond donors enhanced the hydrogen bond interaction between the small molecules and the amino acid residues, further stabilizing the complex structure. Additionally, a higher melting point might reflect stronger internal interactions within PFAAs, and this characteristic might also affect the binding mode to PPARδ. Meanwhile, the docking also revealed that, for isomers with the same molecular formula (such as 3m-PFOA vs. 3,3m_2_-PFOA vs. PFOA), the binding of 3,3m_2_-PFOA with the greatest number of branches was weakest, indicating that the presence of a straight chain affected the binding of the small molecule to the receptor.

When PFAAs interacted with the PPARδ, the binding sites on the protein were mainly concentrated on the amino acid residues TRP-256, TRP-264, LEU-267, ASN-269, and GLY-270. The types of interaction forces were mainly hydrogen bonds and halogen bonds (fluorine atom). The reason for this might be that the strong electronegativity and low polarizability of the fluorine atom lead to strong halogen bond effects. Hydrogen bonds mainly occurred on the carbon–oxygen double bonds, sulfur–oxygen double bonds, and -OH groups of PFAAs. This was one of the key driving forces for the binding of PFAA molecules to PPARδ. This indicated that, when PFAAs interacted with the PPARδ, the terminal substituents on the carbon chain and the length of the carbon chain could affect the types of interaction forces between amino acid residues of PPARδ and PFAAs, as well as the interaction strength between the small molecules and the protein [15,16]. To address the limitations of static docking, further MD simulations were employed to analyze the binding of PFAAs with different molecular weights, and PFAAs with the same molecular weight but different structures and PPARδ. The results demonstrated the dynamic stability of the binding, and confirmed the rules of the influence of molecular weight and branched structure on the binding strength. In the future, it is planned to conduct a study that combines free energy calculations, with the aim of providing more direct and quantitative thermodynamic evidence for the binding affinity between PPARδ and PFAAs.

The results of this study revealed that PFAAs with smaller molecular weight and linear structure should be regarded as a subgroup with higher binding activity. In the risk assessment and prioritization of mixed exposure, more attention should be given to this subgroup. The study confirmed that branching modification is an effective molecular modification to reduce the binding affinity of PPARδ. This provides a computational design idea for the development of safer alternatives to PFAS (such as prioritizing the development of branched variants). Although this study has made certain progress in the analysis of the interaction intensity between PFAAs and PPARδ, there are still certain limitations. This study only employed two types of small molecules, perfluoroalkyl carboxylic acids and perfluoroalkyl sulfonic acids, for molecular docking. However, there are many types of perfluoroalkyl and polyfluoroalkyl substances. The conclusions drawn in this paper cannot be applied to all perfluoroalkyl and polyfluoroalkyl substances. Further research is needed on other types of perfluoroalkyl and polyfluoroalkyl substances.

## Figures and Tables

**Table 1:** Names and acronyms of PFAAs.

Name	Acronym
Pefluoroalkyl Carboxylic Acids (linear)	
Perfluorobutanoic acid	PFBA
perfluorohexanoic acid	PFHxA
perfluoroheptanoic acid	PFHpA
perfluorononanoic acid	PFNA
perfluorodecanoic acid	PFDA
perfluoroundecanoic acid	PFUnDA
perfluorododecanoic acid	PFDoDA
perfluorotetradecanoic acid	PFTeDA
perfluorohexadecanoic acid	PFHxDA
Pefluoroalkyl Carboxylic Acids (Branched)	
perfluoro-3-methylheptanoic acid	3m-PFOA
pefluoro-4-methylheptanoic acid	4m-PFOA
perfluoro-3,3-dimethylhexanoic acid	3,3m_2_-PFOA
perfluoro-4,4-dimethylhexanoic acid	4,4m_2_-PFOA
Perfluoroalkanesulfonic Acids (Linear)	
perfluorobutanesulfonic acid	PFBS
perfluorohexanoic acid	PFHxS
Pefluoroalkyl Carboxylic Acids (Branched)	
perfluoro-1-methylheptanesulfonate	1m-PFOS
perfluoro-2-methylheptanesulfonate	2m-PFOS
perfluoro-3-methylheptanesulfonate	3m-PFOS
perfluoro-4-methylheptanesulfonate	4m-PFOS
perfluoro-5-methylheptanesulfonate	5m-PFOS

**Table 2:** Physicochemical property of PFAAs.

Name	Molecular Formula	CAS NO	Molecular Weight g/mol	Topological Polar Surface Area Å^2^	Boiling Point °C	Number of Hydrogen Bond Receptors	Number of Hydrogen Bond Donors	Density g/mL	Melting Point °C	Flashing Point °C
PFBA	C4HF7O2	375-22-4	214.04	37.3	120	1	9	1.6450	−17.50	120–121
PFHxA	C_6_HF_11_O_2_	307-24-4	314.05	37.3	157	1	13	1.7590	14.00	40.3
PFHpA	C_7_HF_13_O_2_	375-85-9	364.06	37.3	175	1	15	1.7590	30.00	>110
PFNA	C_9_HF_17_O_2_	375-95-1	464.08	37.3	218	1	19	1.7397	59–62	218
PFDA	C_10_HF_19_O_2_	335-76-2	514.08	37.3	218	1	21	1.7490	77–81	218
PFUnDA	C_11_HF_21_O_2_	2058-94-8	564.09	37.3	160	1	23	1.7568	96–101	>110
PFDoDA	C_12_HF_23_O_2_	307-55-1	614.10	37.3	245	1	25	1.7633	105–108	245.00
PFTeDA	C_14_HF_27_O_2_	376-06-7	714.11	37.3	270	1	29	0.8900	130–135	N/A
PFHxDA	C_16_HF_31_O_2_	67905-19-5	814.13	37.3	211	1	33	1.781	154	211
3m-PFOA	C_8_HF_15_O_2_	705240-04-6	414.07	37.3	N/A	1	17	N/A	N/A	N/A
4m-PFOA	C_8_HF_15_O_2_	1144512-18-4	414.07	37.3	N/A	1	17	N/A	N/A	N/A
3,3m_2_-PFOA	C_8_HF_15_O_2_	1812247-20-3	414.07	37.3	N/A	1	17	N/A	N/A	N/A
4,4m_2_-PFOA	C_8_HF_15_O_2_	1192593-79-5	414.07	37.3	N/A	1	17	N/A	N/A	N/A
PFBS	C_4_F_9_SO_3_H	375-73-5	300.10	62.8	112–114	1	12	1.811	N/A	>110
PFHxS	C_6_F_13_SO_3_H	355-46-4	400.11	62.8	238.50	1	16	1.841	N/A	N/A
1m-PFOS	C_8_HF_17_O_3_S	927670-12-0	500.13	62.8	N/A	1	20	N/A	N/A	N/A
2m-PFOS	C_8_HF_17_O_3_S	N/A	500.13	62.8	N/A	1	20	N/A	N/A	N/A
3m-PFOS	C_8_HF_17_O_3_S	N/A	500.13	62.8	N/A	1	20	N/A	N/A	N/A
4m-PFOS	C_8_HF_17_O_3_S	N/A	500.13	62.8	N/A	1	20	N/A	N/A	N/A
5m-PFOS	C_8_HF_17_O_3_S	747385-21-3	500.13	62.8	N/A	1	20	N/A	N/A	N/A

N/A: the data was not available.

**Table 3:** Physicochemical property of PFAAs in validation group.

Name	Molecular Formula	CAS NO	Molecular Weight g/mol	Topological polar Surface Area Å^2^	Boiling Point °C	Number of Hydrogen Bond Receptors	Number of Hydrogen Bond Donors	Density g/mL	Melting Point °C	Flashing Point °C
PFPA	C_5_HF_9_O_2_	2706-90-3	264.05	37.3	140	1	11	1.7130	N/A	140
PFOA	C_8_HF_15_O_2_	335-67-1	414.07	37.3	189	1	17	1.7	55–56	189–192
5m-PFOA	C_8_HF_15_O_2_	909009-42-3	414.07	37.3	N/A	1	17	N/A	N/A	N/A
PFOS	C_8_F_17_SO_3_H	1763-23-1	500.13	62.8	260	1	20	1.8 ± 0.1	90.00	11.00
6m-PFOS	C_8_F_17_SO_3_H	255831-20-0	500.13	62.8	N/A	1	20	N/A	N/A	N/A

N/A: the data was not available.

**Table 4:** The binding data of PFBA, 3m-PFOA, PFBS, and 1m-PFOS with PPARδ.

Compound	Amino Acid Residue	Binding Site of Small Molecule	Type of Interaction Force	Length of Chemical Bond (Å)
PFBA	TRP-256	—OH	Hydrogen bond	2.23
	ASN-269	—OH	Hydrogen bond	2.03
		C=O	Hydrogen bond	2.05
	GLY-270	—OH	Hydrogen bond	1.95
	GLU-276	—F	Halogen bond	2.64
				3.69
3m-PFOA	TRP-256	—OH	Hydrogen bond	3.05
	TRP-264	—F	Halogen bond	3.05
	LEU-267	—F	Halogen bond	2.94
	ASN-269	—OH	Hydrogen bond	2.91
	GLY-270	—OH	Hydrogen bond	2.98
		—OH	Hydrogen bond	2.87
PFBS	TRP-256	—OH	Hydrogen bond	3.05
	TRP-264	—F	Halogen bond	3.05
	LEU-267	—F	Halogen bond	2.94
	ASN-269	—OH	Hydrogen bond	2.91
	GLY-270	—OH	Hydrogen bond	2.87
		S=O	Hydrogen bond	2.98
1m-PFOS	TRP-256	—OH	Hydrogen bond	3.12
	VAL-263	—F	Halogen bond	3.99
	TRP-264	—F	Halogen bond	3.82
		—F	Halogen bond	3.82
	ASN-269	—OH	Hydrogen bond	3.17
	GLY-270	—OH	Hydrogen bond	2.70
		S=O	Hydrogen bond	3.18
	GLU-276	S=O	Hydrogen bond	4.08

**Table 5:** Pearson correlation and Spearman correlation analysis of interaction strength of PFAAs with PPARδ and physicochemical properties of PFAAs.

Physicochemical Property	Pearson Correlation		Spearman Correlation	
	Correlation Coefficient	*p*	Correlation Coefficient	*p*
Molecular weight	0.581 **	0.007	0.453 *	0.045
Number of hydrogen bond receptors	b		b	.
Number of hydrogen bond donors	0.608 **	0.004	0.453 *	0.045
Topological polar surface area	−0.738 **	<0.01	−0.809 **	<0.001
Density	−0.509	0.11	−0.497	0.12
Melting point	0.866 **	0.003	0.883 **	0.002
Boiling point	0.675 *	0.023	0.579	0.062
Flash point	0.643	0.062	0.707 *	0.033

** Significant at the 0.01 level; * Significant at the 0.05 level; b Correlation cannot be calculated as at least one variable is constant.

**Table 6:** Analysis of the interaction strength of PFAAs in the verification group with PPARδ.

Name	Binding Energy (kcal·mol^−1^)	Molecular Weight (g·mol^−1^)	Number of Hydrogen Bond Donos	Melting Point (°C)
PFPA	−3.07	264.05	11	N/A
PFOA	−3.89	414.07	17	55–56
5m-PFOA	−3.83	414.07	17	N/A
PFOS	−3.53	500.13	20	90.00
6m-PFOS	−3.56	500.13	20	N/A

N/A: the data was not available.

**Table 7:** The key information of four small molecules used for MD simulations.

Name	Molecular Weight	Molecular Formula	Presence of Branched Structures (Y/N)	Range of RMSD	Binding Energy (kcal·mol^−1^)
PFBA	214.04	C4HF7O2	N	0.0004978~0.2577104	−2.99
PFOA	414.07	C_8_HF_15_O_2_	N	0.0004996~0.3649920	−3.09
3,3m_2_-PFOA	414.07	C_8_HF_15_O_2_	Y	0.000500~0.442259	−2.45
PFHxA	814.13	C_16_HF_31_O_2_	N	0.0005057~0.3229235	−1.50

## Data Availability

The original contributions presented in this study are included in the article/Supplementary Material. Further inquiries can be directed to the corresponding authors.

## Supplementary Materials

The following supporting information can be downloaded at https://www.mdpi.com/article/10.3390/toxics14010067/s1. Figure S1: The docking interaction results of PFHxA and PPARδ; Figure S2: The docking interaction results of PFHpA and PPARδ; Figure S3: The docking interaction results of PFHpA and PPARδ; Figure S4: The docking interaction results of PFDA and PPARδ; Figure S5: The docking interaction results of PFUnDA and PPARδ; Figure S6: The docking interaction results of PFDoDA and PPARδ; Figure S7: The docking interaction results of PFTeDA and PPARδ; Figure S8. The docking interaction results of PFHxDA and PPARδ; Figure S9: The docking interaction results of 4m-PFOA and PPARδ; Figure S11: The docking interaction results of 4,4m2-PFOA and PPARδ; Figure S12: The docking interaction results of PFHxS and PPARδ; Figure S13: The docking interaction results of 2m-PFOS and PPARδ; Figure S14: The docking interaction results of 3m-PFOS and PPARδ; Figure S15: The docking interaction results of 4m-PFOS and PPARδ; Figure S16: The docking interaction results of 5m-PFOS and PPARδ; Figure S17. High-frequency amino acid distribution map of PFAAs in validation group: 1. PFPA; 2. PFOA; 3. 5m-PFOA; 4. PFOS; 5. 6m-PFOS; Table S1: The binding energies and docking scores of all PFAAs with PPARδ; Table S2: The binding data of PFBA and PPARδ; Table S3: The binding data of PFHpA and PPARδ; Table S4: The binding data of PFNA and PPARδ; Table S5: The binding data of PFDAand PPARδ; Table S6: The binding data of PFUnDA and PPARδ; Table S7: The binding data of PFDoDA and PPARδ; Table S8: The binding data of PFTeDA and PPARδ; Table S9: The binding data of PFHxDA and PPARδ; Table S10: The binding data of 4m-PFOA and PPARδ; Table S11. The binding data of 3,3m2-PFOA and PPARδ; Table S12. The binding data of 4,4m2-PFOA and PPARδ; Table S13: The binding data of PFHxS and PPARδ; Table S14. The binding data of 2m-PFOS and PPARδ; Table S15: The binding data of 3m-PFOS and PPARδ; Table S16: The binding data of 4m-PFOS and PPARδ; Table S17: The binding data of 5m-PFOS and PPARδ; Table S18. Statistics of amino acid residues of PPARδ interacting with PFAAs; Table S19. Physicochemical properties of PFAAs in the validation group; Table S20: The binding data of PFPA and PPARδ; Table S21. The binding data of PFOA and PPARδ; Table S22: The binding data of 5m-PFOA and PPARδ; Table S23. The binding data of PFOS and PPARδ; Table S24: The binding data of 6m-PFOS and PPARδ.
