# Supplementary material for: Study on the Molecular Mechanism of Interaction Between Perfluoroalkyl Acids and PPAR by Molecular Docking
Source: Toxics. 2026 Jan 11;14(1):67. doi: 10.3390/toxics14010067 (PMC12846299; doi:10.3390/toxics14010067)
Supplement: Supplementary file 1 [file toxics-14-00067-s001.zip › toxics-4007459-supplementary.pdf]

Table S1. The binding energies and docking scores of all PFAAs with PPARδ

PFAAs	Binding energy (kcal/mol)	Cluster RMSD	Reference RMSD
PFBA	-2.99	0.00	69.44
PFPA	-3.07	0.00	68.11
PFHxA	-3.22	0.00	66.65
PFHpA	-3.06	0.00	67.39
PFOA	-3.09	0.00	32.48
PFNA	-2.83	0.00	66.18
PFDA	-1.91	0.00	64.21
PFUnDA	-2.70	0.00	64.34
PFDoDA	-2.07	0.00	66.52
PFTeDA	-1.30	0.00	66.30
PFHxDA	-1.50	0.00	65.85
3m-PFOA	-2.92	0.00	68.06
4m-PFOA	-3.55	0.00	68.27
5m-PFOA	-3.83	0.00	66.20
3,3m$_2$-PFOA	-2.45	0.00	68.35
4,4m$_2$-PFOA	-2.71	0.00	67.04
PFBS	-4.78	0.00	68.44
PFHxS	-3.61	0.00	67.04
PFOS	-3.53	0.00	64.14
1m-PFOS	-3.65	0.00	67.10
2m-PFOS	-3.32	0.00	66.42
3m-PFOS	-4.50	0.00	67.36
4m-PFOS	-3.77	0.00	66.10
5m-PFOS	-3.83	0.00	66.95
6m-PFOS	-3.56	0.00	66.36

The binding mode of perfluoroalkyl carboxylic acids (straight chain) with PPAR δ

The docking results and binding data of PFHxA binding to PPAR δ were shown in SI Figure 1 and SI Table 2. The key amino acid residues for the interaction between PFHxA and PPAR δ were TRP-256, ASN-269, GLY-270, GLU-276, and TRP-246. The binding energy is -3.22 kcal·mol^{-1}.

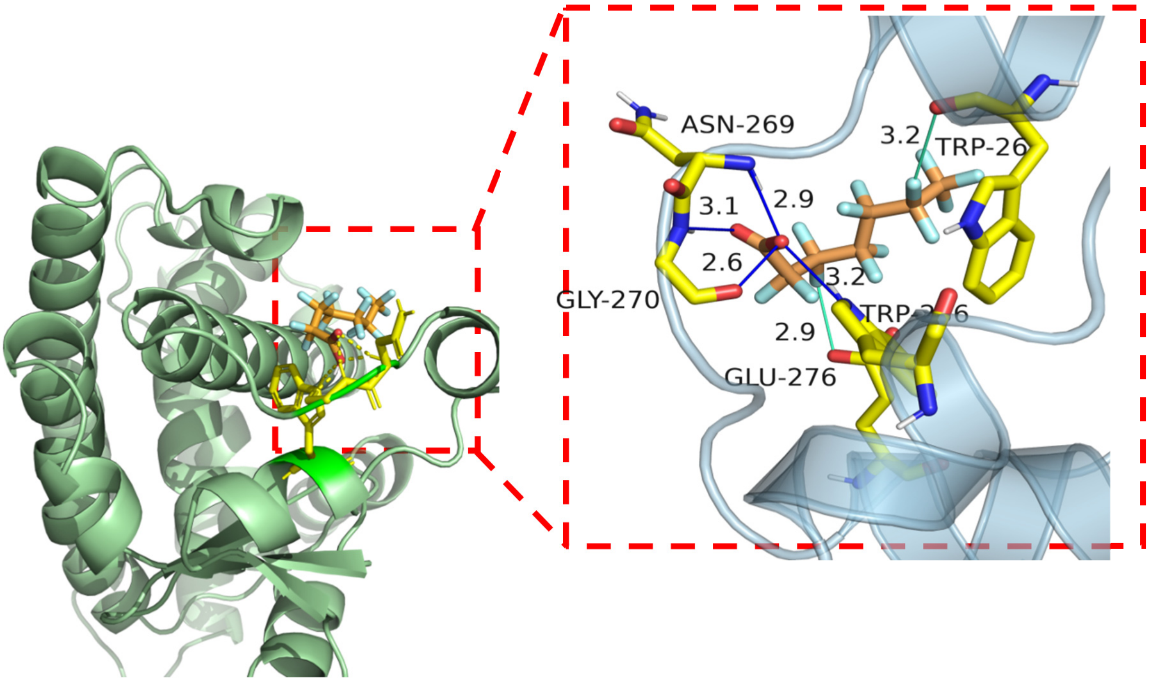

Figure S1. The docking interaction results of PFHxA and PPAR δ

Table S2. The binding data of PFBA and PPAR δ

Amino acid residue	Binding site of small molecule	Type of interaction force	Length of chemical bond（Å）
TRP-256	—OH	Hydrogen bond	2.25
TRP-264	—F	Halogen bond	3.24
ASN-269	—OH	Hydrogen bond	1.98
GLY-270	C=O	Hydrogen bond	1.66
	—OH	Hydrogen bond	2.12
GLU-276	—F	Halogen bond	2.69

The binding data and docking results of PFHpA with PPAR δ were shown in SI Table 3 and SI Figure 2. The key amino acid residues involved in the interaction between PFHpA and PPAR δ were TRP-256, ASN-269, GLY-270, GLU-276, TRP-264, and LEU-267, with the lowest binding energy being -3.06 kcal·mol^{-1}.

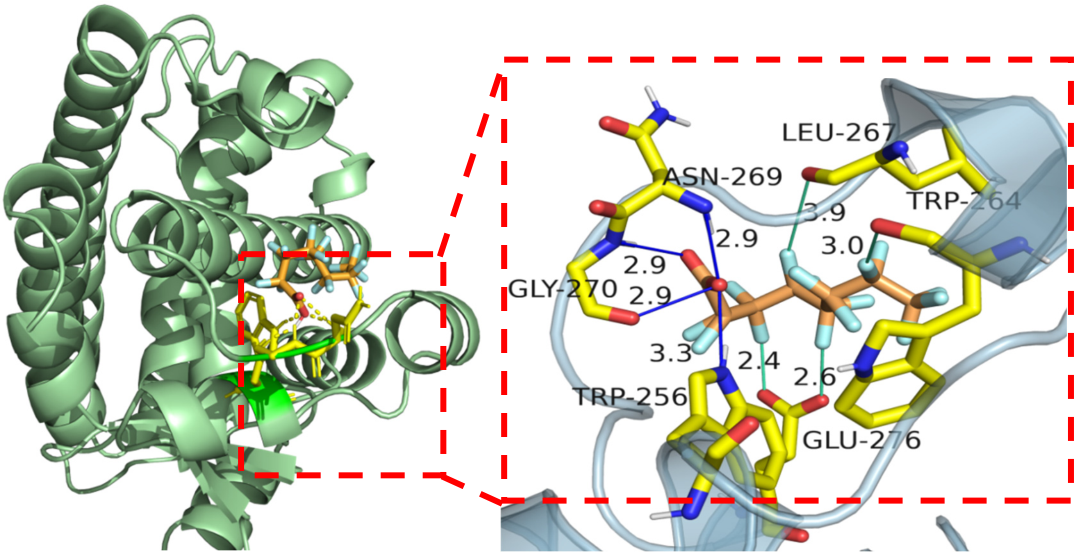

Figure S2. The docking interaction results of PFHpA and PPAR δ

Table S3. The binding data of PFHpA and PPAR δ

Amino acid residue	Binding site of small molecule	Type of interaction force	Length of chemical bond（Å）
TRP-256	—OH	Hydrogen bond	2.28
TRP-264	—F	Halogen bond	2.97
LEU-267	—F	Halogen bond	3.89
ASN-269	—OH	Hydrogen bond	1.89
GLY-270	C=O	Hydrogen bond	2.00
	—OH	Hydrogen bond	1.95
GLU-276	—F	Halogen bond	2.44
			2.62

The binding data and docking results of PFNA and PPAR δ were shown in SI Table 4 and SI Figure 3. The key amino acids involved in the interaction between PFNA and PPAR δ were TRP-256, ASN-269, GLY-270, GLU-276, and TRP-264, with the lowest binding energy being -2.83 $kcal \cdot mol^{-1}$.

Table S4. The binding data of PFNA and PPAR δ

Amino acid residue	Binding site of small molecule	Type of interaction force	Length of chemical bond（Å）
TRP-256	—OH	Hydrogen bond	2.16
TRP-264	—F	Halogen bond	3.93
	—F	Halogen bond	3.90
ASN-269	—OH	Hydrogen bond	2.16
GLY-270	C=O	Hydrogen bond	1.94
	—OH	Hydrogen bond	2.12

GLU-276	—F	Halogen bond	3.08

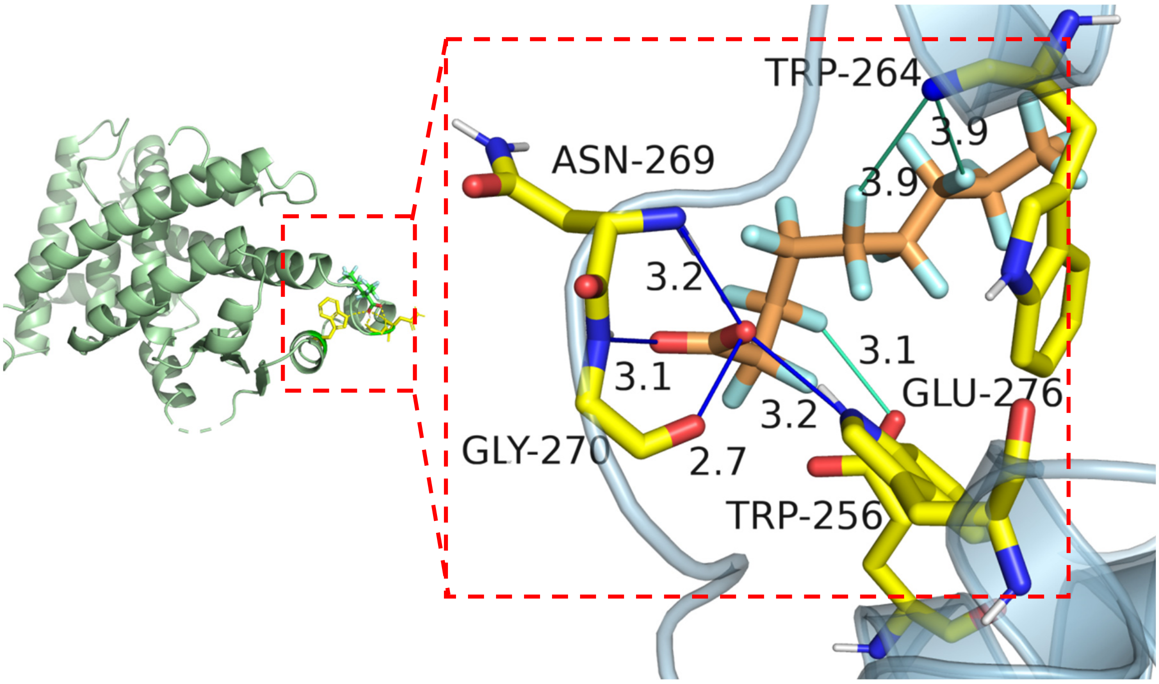

Figure S3. The docking interaction results of PFHpA and PPAR δ

The binding data and docking results of PFDA with PPAR δ were shown in SI Table 5 and SI Figure 4. The key amino acids for the interaction between PFDA and PPAR δ were TRP-256, TRP-264, ASN-269, GLY-270, and GLU-276. The lowest binding energy was -1.91 kcal·mol^{-1}.

Table S5. The binding data of PFDAand PPAR δ

Amino acid residue	Binding site of small molecule	Type of interaction force	Length of chemical bond（Å）
TRP-256	—OH	Hydrogen bond	3.58
TRP-264	—F	Halogen bond	3.63
ASN-269	—OH	Hydrogen bond	2.78
GLY-270	C=O	Hydrogen bond	3.10
	—OH	Hydrogen bond	2.74
GLU-276	—F	Halogen bond	3.67

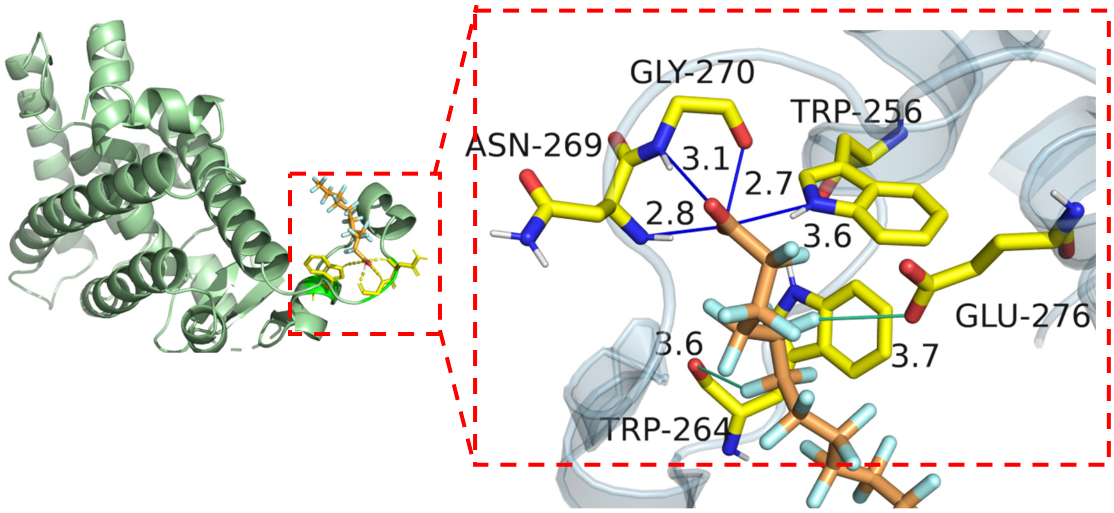

Figure S4. The docking interaction results of PFDAand PPAR δ

The binding data and docking results of PFUnDA with PPAR δ were shown in SI Table 6 and SI Figure 5. The key amino acids for the interaction between PFUnA and PPAR δ were TRP-256, TRP-264, ASN-269, and GLY-270. The lowest binding energy was -2.70 kcal·mol^{-1}.

Table S6. The binding data of PFUnDA and PPAR δ

Amino acid residue	Binding site of small molecule	Type of interaction force	Length of chemical bond（Å）
TRP-256	—OH	Hydrogen bond	3.27
LEU-267	—F	Halogen bond	3.64
ASN-269	—OH	Hydrogen bond	3.02
GLY-270	C=O	Hydrogen bond	3.10
	—OH	Hydrogen bond	2.46

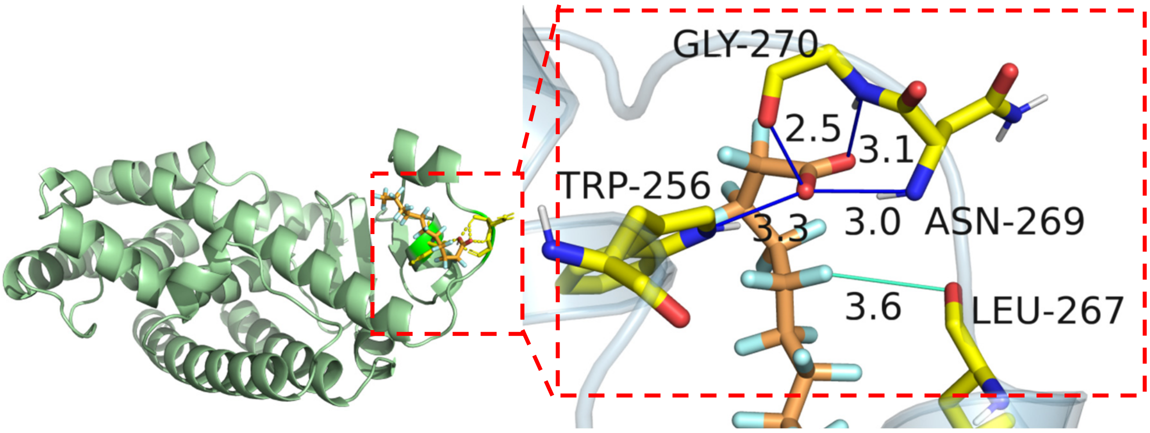

Figure S5. The docking interaction results of PFUnDA and PPAR δ

The binding data and docking results of PFDoDA with PPARδ were shown in SI Table 7 and SI Figure 6. The key amino acids for the interaction between PFDoDA and PPARδ were TRP-256, TRP-264, ASN-269, GLY-270, and GLU276. The lowest binding energy was -2.07 kcal·mol^{-1}.

Table S7. The binding data of PFDoDA and PPARδ

Amino acid residue	Binding site of small molecule	Type of interaction force	Length of chemical bond（Å）
TRP-256	—OH	Hydrogen bond	2.89
TRP-264	—F	Halogen bond	3.81
ASN-269	—OH	Hydrogen bond	3.08
GLY-270	C=O	Hydrogen bond	3.99
	—OH	Hydrogen bond	2.75
GLU-276	—F	Halogen bond	3.08

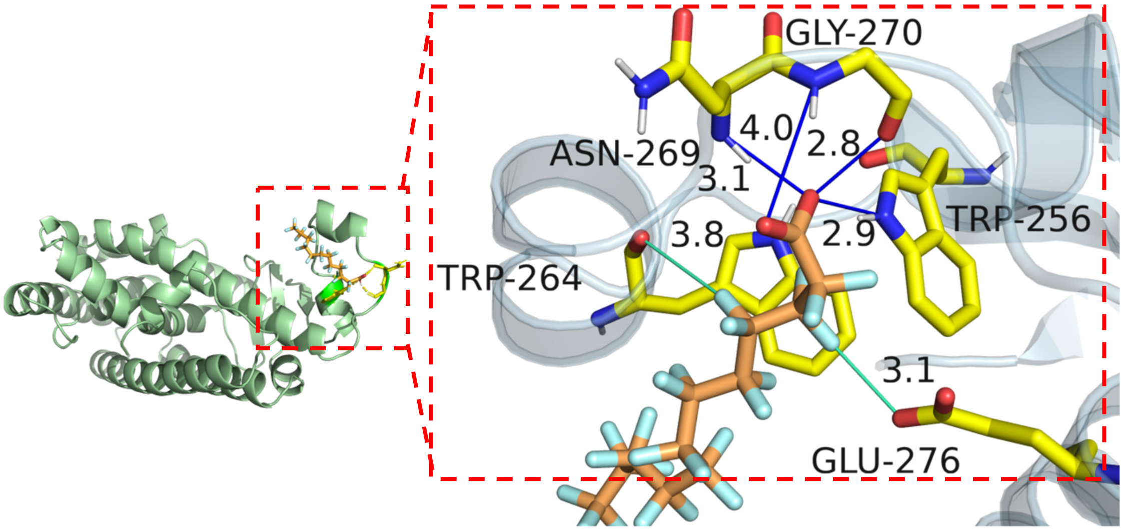

Figure S6. The docking interaction results of PFDoDA and PPARδ

The binding data and docking results of PFTeDA with PPARδ were shown in SI Table 8 and SI Figure 7. The key amino acids for the interaction between PFTeDA and PPARδ were GLU-276, GLY-270, and HIS-280, and the lowest binding energy was -1.30 kcal·mol^{-1}.

Table S8. The binding data of PFTeDA and PPARδ

Amino acid residue	Binding site of small molecule	Type of interaction force	Length of chemical bond（Å）
GLY-270	—F	Halogen bond	3.23
GLU-276	—F	Halogen bond	2.99

	—OH	Hydrogen bond	2.98
HIS-280	terminal carbon	Salt bridge	3.86

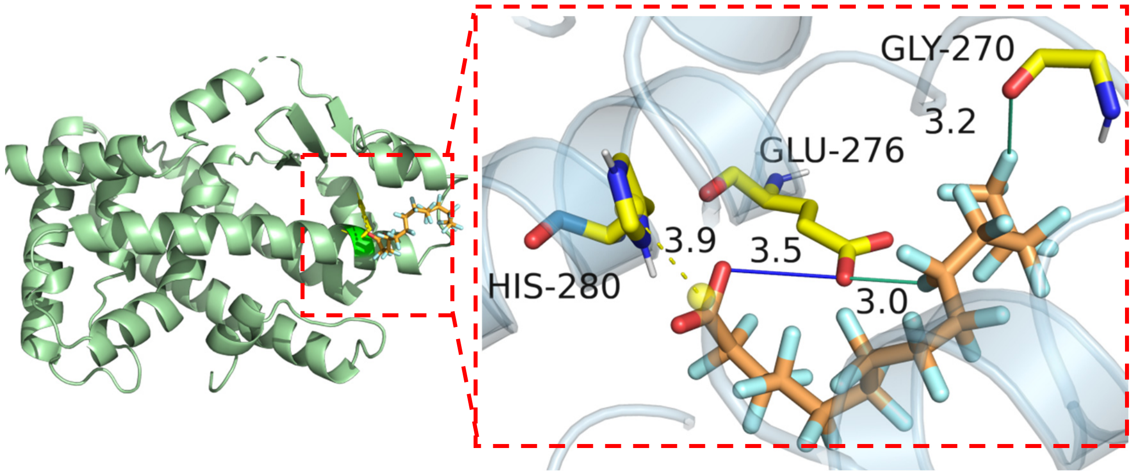

Figure S7. The docking interaction results of PFTeDA and PPAR δ

The binding data and docking results of PFHxDA with PPAR δ were shown in SI Table 9 and Figure 8. The key amino acids involved in the interaction between PFHxDA and PPAR δ were LEU-267, GLU-276, and HIS-280, with the lowest binding energy being -1.50 kcal·mol^{-1}.

Table S9. The binding data of PFHxDA and PPAR δ

Amino acid residue	Binding site of small molecule	Type of interaction force	Length of chemical bond（Å）
LEU-267	—F	Halogen bond	2.75
GLU-276	—OH	Hydrogen bond	2.02
HIS-280	terminal carbon	Salt bridge	5.14

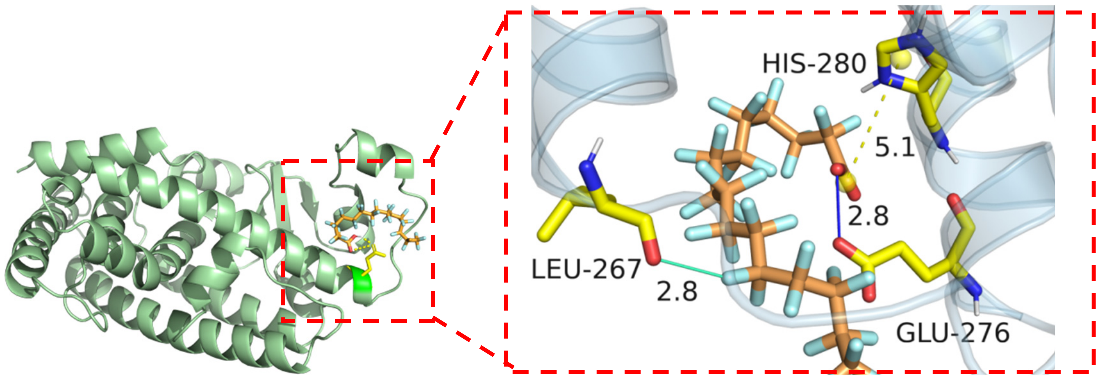

Figure S8. The docking interaction results of PFHxDAand PPAR δ

Pefluoroalkyl Carboxylic Acids (Branched)

The binding data and docking results of 4m-PFOA with PPAR δ were shown in SI

Table 10 and SI Figure 9. The key amino acids involved in the interaction between 4m-PFOA and PPARδ were TRP-256, ASN-269, GLY-270, and VAL-263, with the lowest binding energy being -3.55 kcal·mol^{-1}.

Table S10. The binding data of 4m-PFOA and PPARδ

Amino acid residue	Binding site of small molecule	Type of interaction force	Length of chemical bond（Å）
TRP-256	—OH	Hydrogen bond	3.23
VAL-263	—F	Halogen bond	3.87
ASN-269	—OH	Hydrogen bond	3.01
GLY-270	—OH	Hydrogen bond	2.90
	C=O	Hydrogen bond	2.64

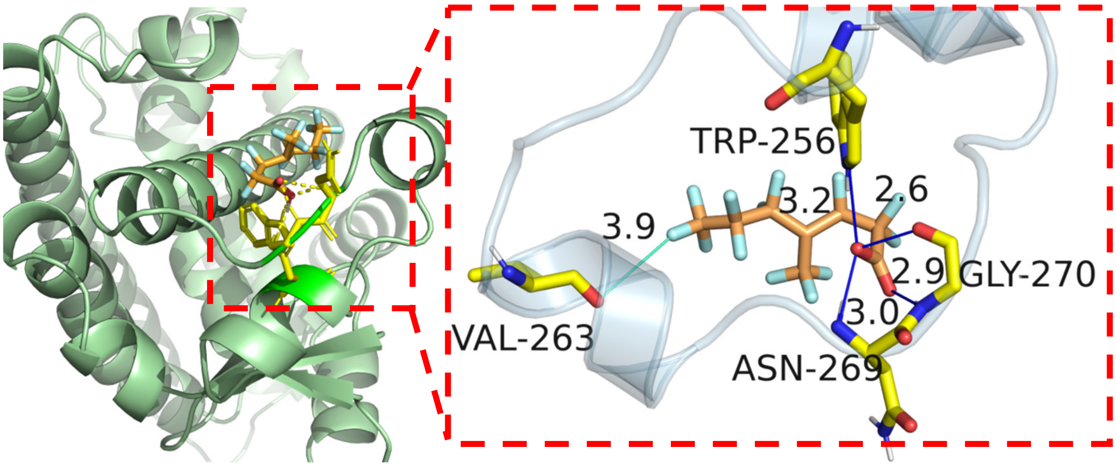

Figure S9. The docking interaction results of 4m-PFOA and PPARδ

The binding data and docking results of 3,3m$_2$-PFOA with PPARδ were shown in SI Table 11 and SI Figure 10. The key amino acids involved in the interaction between 3,3m$_2$-PFOA and PPARδ were TRP-256, TRP-264, LEU-267, ASN-269, GLY-270, and GLU-279, with the lowest binding energy being -2.45 kcal·mol^{-1}.

Table S11. The binding data of 3,3m$_2$-PFOA and PPARδ

Amino acid residue	Binding site of small molecule	Type of interaction force	Length of chemical bond（Å）
TRP-256	—OH	Hydrogen bond	3.16
TRP-264	—F	Halogen bond	3.17
LEU-267	—F	Halogen bond	3.10
ASN-269	—OH	Hydrogen bond	3.05
GLY-270	—OH	Hydrogen bond	2.92
	C=O	Hydrogen bond	2.97
GLU-279	—F	Halogen bond	3.49
	—F	Halogen bond	3.34

	—F	Halogen bond	2.76

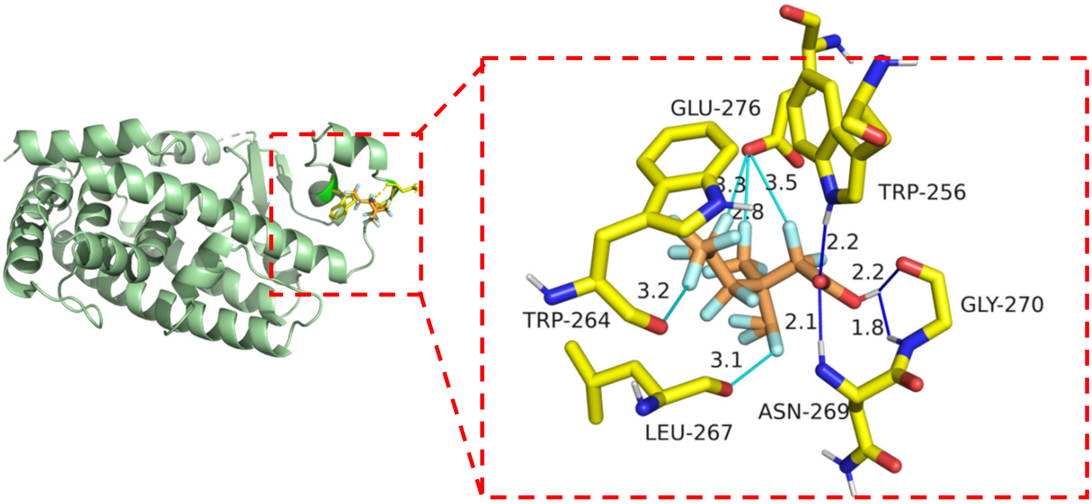

Figure S10. The docking interaction results of 3,3m2-PFOA and PPAR δ

The docking results of 4,4m2-PFOA with PPAR δ were shown in SI Table 12 and SI Figure 11. The key amino acids for the interaction between 4,4m2-PFOA and PPAR δ were TRP-256, TRP-264, LEU-267, ASN-269, and GLY-270. The lowest binding energy was -2.71 kcal·mol^{-1}.

Table S12. The binding data of 4,4m2-PFOA and PPAR δ

Amino acid residue	Binding site of small molecule	Type of interaction force	Length of chemical bond（Å）
TRP-256	—OH	Hydrogen bond	2.11
TRP-264	—F	Halogen bond	2.75
LEU-267	—F	Halogen bond	3.91
	—F	Halogen bond	2.80
ASN-269	—OH	Hydrogen bond	2.07
GLY-270	—OH	Hydrogen bond	3.10
	C=O	Hydrogen bond	2.02

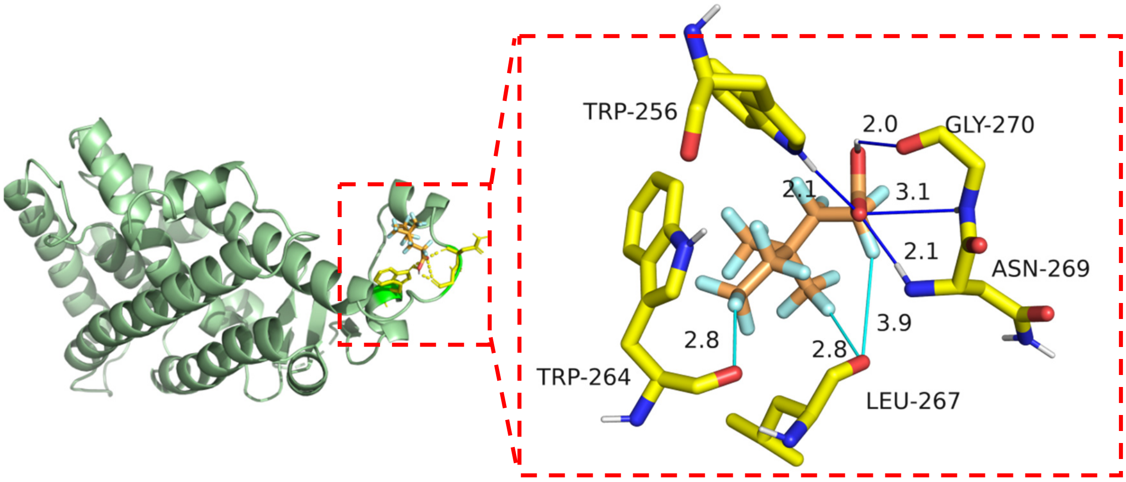

Figure S11. The docking interaction results of 4,4m2-PFOA and PPAR δ

Perfluoroalkanesulfonic Acids (Linear)

The binding data and docking results of PFHxS with PPAR δ were shown in SI Table 13 and SI Figure 12. The key amino acids for the interaction between PFHxS and PPAR δ were TRP-256, LEU-267, ASN-269, GLY-270, and GLU-276. The lowest binding energy was -3.61 kcal·mol^{-1}.

Table S13. The binding data of PFHxS and PPAR δ

Amino acid residue	Binding site of small molecule	Type of interaction force	Length of chemical bond（Å）
TRP-256	—OH	Hydrogen bond	2.87
LEU-267	—F	Halogen bond	3.21
ASN-269	—OH	Hydrogen bond	3.24
GLY-270	—OH	Hydrogen bond	3.45
	S=O	Hydrogen bond	2.50
GLU-276	—F	Halogen bond	3.38
	—F	Halogen bond	2.83

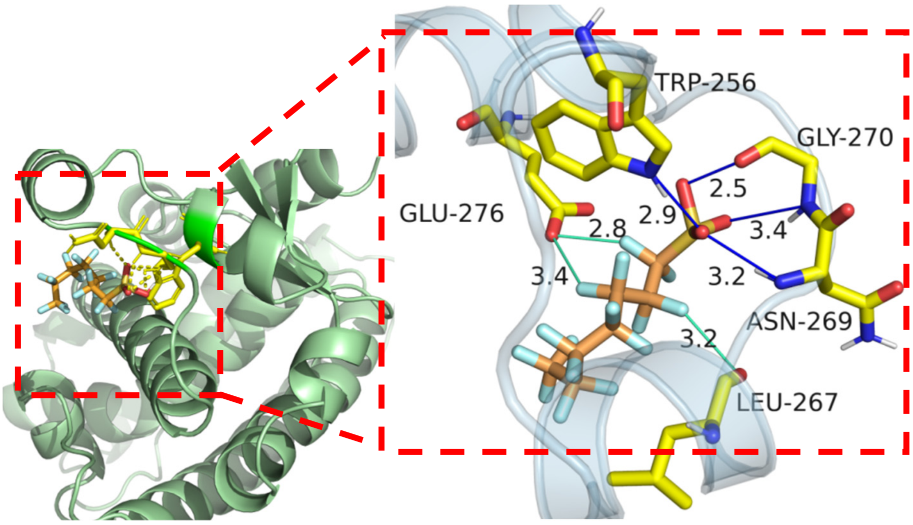

Figure S12. The docking interaction results of PFHxS and PPAR δ

Pefluoroalkyl Carboxylic Acids (Branched)

The binding data and docking results of 2m-PFOS with PPAR δ were shown in SI Table 14 and SI Figure 13. The key amino acids for the interaction between 2m-PFOS and PPAR δ were TRP-256, LEU-267, ASN-269, and GLY-270, and the lowest binding energy was -3.32 kcal·mol^{-1}.

Table S14. The binding data of 2m-PFOS and PPAR δ

Amino acid residue	Binding site of small molecule	Type of interaction force	Length of chemical bond（Å）
TRP-256	—OH	Hydrogen bond	3.12
LEU-267	S=O	Hydrogen bond	3.74
ASN-269	—OH	Hydrogen bond	2.81
GLY-270	S=O	Hydrogen bond	4.09
	—OH	Hydrogen bond	2.58

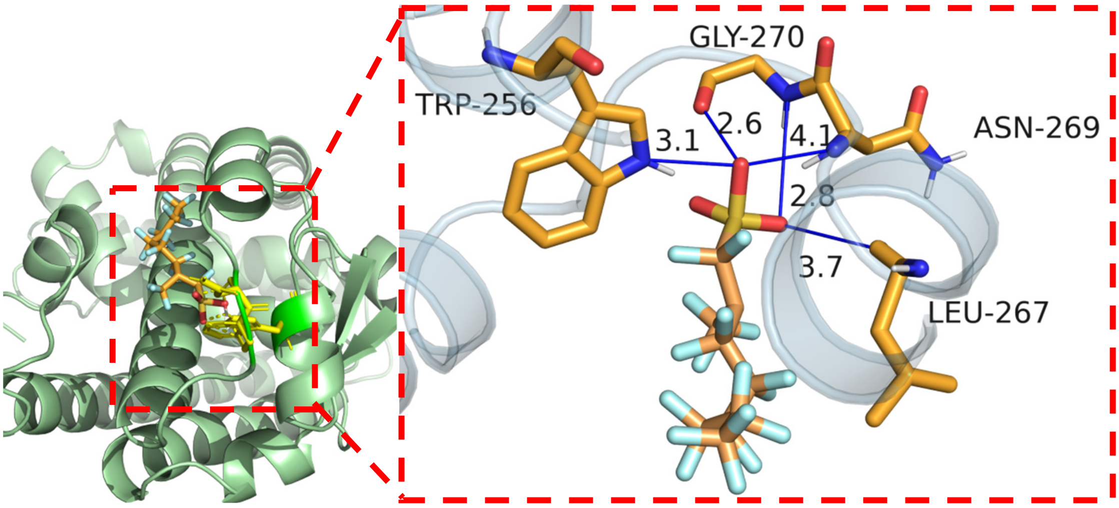

Figure S13. The docking interaction results of 2m-PFOS and PPAR δ

The binding data and docking results of 3m-PFOS with PPAR δ were shown in SI Table 15 and SI Figure 14. The key amino acids involved in the interaction between 3m-PFOS and PPAR δ were TRP-256, TRP-264, ASN-269, GLY-270, and GLU-276. The lowest binding energy was -4.50 kcal·mol^{-1}.

Table S15. The binding data of 3m-PFOS and PPAR δ

Amino acid residue	Binding site of small molecule	Type of interaction force	Length of chemical bond（Å）
TRP-256	—OH	Hydrogen bond	3.05
TRP-264	—F	Halogen bond	3.24
LEU-267	—F	Halogen bond	4.00
ASN-269	—OH	Hydrogen bond	2.94
GLY-270	S=O	Hydrogen bond	2.92
	—OH	Hydrogen bond	2.84
GLU-276	—F	Halogen bond	3.99

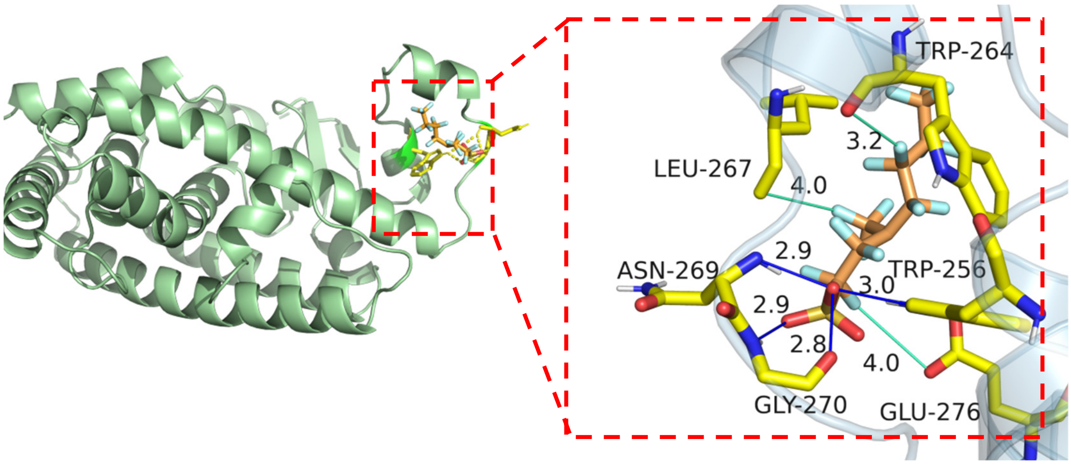

Figure S14. The docking interaction results of 3m-PFOS and PPAR δ

The binding data and docking results of 4m-PFOS with PPAR δ were shown in SI Table 16 and SI Figure 15. The key amino acids involved in the interaction between 4m-PFOS and PPAR δ were TRP-256, TRP-264, ASN-269, GLY-270, and GLU-276. The lowest binding energy was -3.77 kcal·mol^{-1}.

Table S16. The binding data of 4m-PFOS and PPAR δ

Amino acid residue	Binding site of small molecule	Type of interaction force	Length of chemical bond（Å）
TRP-256	—OH	Hydrogen bond	2.99
TRP-264	—F	Halogen bond	3.65
ASN-269	—OH	Hydrogen bond	3.00
GLY-270	S=O	Hydrogen bond	2.70

	—OH	Hydrogen bond	2.80
GLU-276	—F	Halogen bond	3.74

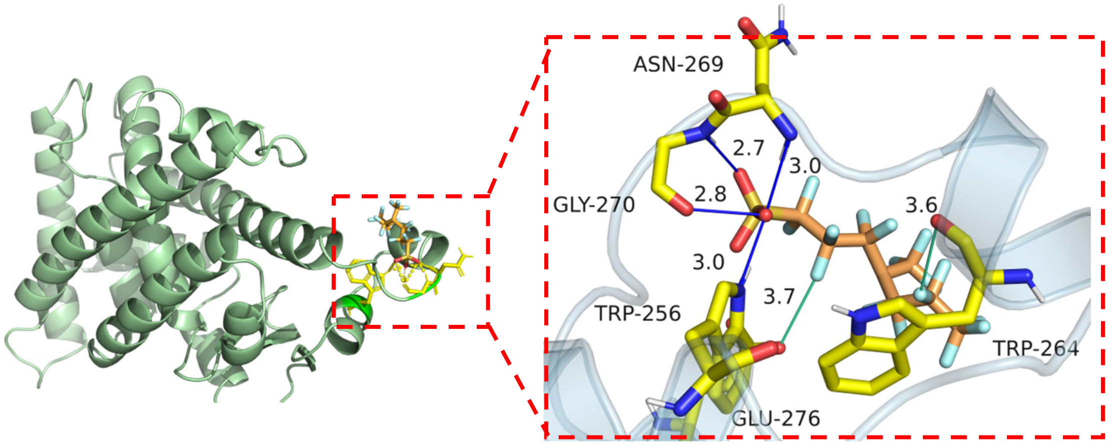

Figure S15. The docking interaction results of 4m-PFOS and PPAR δ

The binding data and docking results of 5m-PFOS with PPAR δ were shown in SI Table 17 and SI Figure 16. The key amino acids involved in the interaction between 5m-PFOS and PPAR δ were TRP-256, LEU-267, ASN-269, GLY-270, and GLU-276. The lowest binding energy was -3.83 kcal·mol^{-1}.

Table S17. The binding data of 5m-PFOS and PPAR δ

Amino acid residue	Binding site of small molecule	Type of interaction force	Length of chemical bond （Å）
TRP-256	—OH	Hydrogen bond	3.02
LEU-267	—F	Halogen bond	2.86
ASN-269	—OH	Hydrogen bond	2.87
GLY-270	S=O	Hydrogen bond	3.36
	—OH	Hydrogen bond	2.90
GLU-276	—F	Halogen bond	3.67

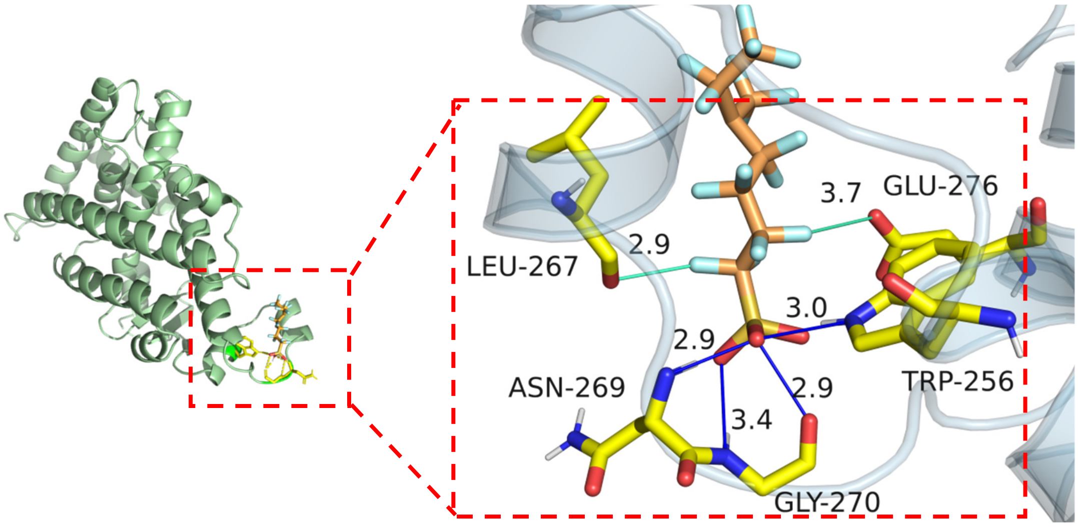

Figure S16. The docking interaction results of 5m-PFOS and PPAR δ

Table S18. Statistics of amino acid residues of PPAR δ interacting with PFAAs

PFAAs	Amino acid residues of PPAR δ
PFBA	TRP-256、ASN-269、GLY-270、GLU276
PFHxA	TRP-246、TRP-256、ASN-269、GLY-270、GLU-276
PFHpA	TRP-256、TRP-264、LEU-267、ASN-269、GLY-270、GLU-276
PFNA	TRP-256、TRP-264、ASN-269、GLY-270、GLU-276
PFDA	TRP-256、TRP-264、ASN-269、GLY-270、GLU-276
PFUnDA	TRP-256、TRP-264、ASN-269、GLY-270、GLU-276
PFDoDA	TRP-256、LEU-267、ASN-269、GLY-270
PFTeDA	GLY-270、GLU-276、HIS-280
PFHxDA	LEU-267、GLU-276、HIS-280
3m-PFOA	TRP-256、ASN-269、GLY-270、GLU-276
4m-PFOA	TRP-256、VAL-263、ASN-269、GLY-270
3,3m$_2$-PFOA	TRP-256、TRP-264、LEU-267、ASN-269、GLY-270、GLU-279
4,4m$_2$-PFOA	TRP-256、TRP-264、LEU-267、ASN-269、GLY-270
PFBS	TRP-256、TRP-264、LEU-267、ASN-269、GLY-270
PFHxS	TRP-256、LEU-267、ASN-269、GLY-270、GLU-276
1m-PFOS	TRP-256、VAL-263、TRP-264、ASN-269、GLY-270、GLU-276
2m-PFOS	TRP-256、LEU-267、ASN-269、GLY-270、GLU-276
3m-PFOS	TRP-256、TRP-264、ASN-269、GLY-270、GLU-276
4m-PFOS	TRP-256、TRP-264、ASN-269、GLY-270、GLU-276
5m-PFOS	TRP-256、LEU-267、ASN-269、GLY-270、GLU-276

Table S19. Physicochemical properties of PFAAs in the validation group

Name	Molecular formula	CAS NO.	Molecular weight g/mol	Topological polar surface area Å²	Boiling point ℃
PFPA	$C_5HF_9O_2$	2706-90-3	264.05	37.3	140
PFOA	$C_8HF_{15}O_2$	335-67-1	414.07	37.3	189
5m-PFOA	$C_8HF_{15}O_2$	909009-42-3	414.07	37.3	N/A
PFOS	$C_8F_{17}SO_3H$	1763-23-1	500.13	62.8	260
6m-PFOS	$C_8F_{17}SO_3H$	255831-20-0	500.13	62.8	N/A

Name	Number of hydrogen bond acceptors	Number of hydrogen bond receptors	Density g/mL	Melting point ℃	Flashing point ℃

PFPA	1	11	1.7130	N/A	140
PFOA	1	17	1.7	55-56	189-192
5m-PFOA	1	17	N/A	N/A	N/A
PFOS	1	20	1.8±0.1	90.00	11.00
6m-PFOS	1	20	N/A	N/A	N/A

N/A :the data was not found.

Table S20. The binding data of PFPA and PPAR δ

Amino acid residue	Binding site of small molecule	Type of interaction force	Length of chemical bond（Å）
TRP-256	—OH	Hydrogen bond	3.21
TRP-264	—F	Halogen bond	3.02
LEU-267	—F	Halogen bond	2.52
ASN-269	—OH	Hydrogen bond	2.89
GLY-270	—OH	Hydrogen bond	3.42
	—OH	Hydrogen bond	2.74
GLU-276	—F	Halogen bond	3.68

Table S21. The binding data of PFOA and PPAR δ

Amino acid residue	Binding site of small molecule	Type of interaction force	Length of chemical bond（Å）
LEU-211	C=O	Hydrogen bond	2.81
LYS-212	—F	Halogen bond	2.88
	—OH	Hydrogen bond	2.87
ALA-213	—OH	Hydrogen bond	2.78

Table S22. The binding data of 5m-PFOA and PPAR δ

Amino acid residue	Binding site of small molecule	Type of interaction force	Length of chemical bond（Å）
TRP-256	—OH	Hydrogen bond	3.02
LEU-267	—F	Halogen bond	2.86
ASN-269	—OH	Hydrogen bond	2.87
GLY-270	—OH	Hydrogen bond	2.90
	C=O	Hydrogen bond	3.36
GLU-276	—F	Halogen bond	3.67

Table S23. The binding data of PFOS and PPAR δ

Amino acid residue	Binding site of small molecule	Type of interaction force	Length of chemical bond（Å）

TRP-256	—OH	Hydrogen bond	3.09
LEU-267	S=O	Hydrogen bond	3.86
ASN-269	S=O	Hydrogen bond	2.90
GLY-270	—OH	Hydrogen bond	2.82
	S=O	Hydrogen bond	3.94

Table S24. The binding data of 6m-PFOS and PPAR δ

Amino acid residue	Binding site of small molecule	Type of interaction force	Length of chemical bond（Å）
TRP-256	—F	Halogen bond	2.85
TRP-264	—F	hydrophobic interaction	3.62
LEU-267	S=O	Hydrogen bond	3.53
ASN-269	—OH	Hydrogen bond	3.06
GLY-270	—OH	Hydrogen bond	2.83
	S=O	Hydrogen bond	3.84

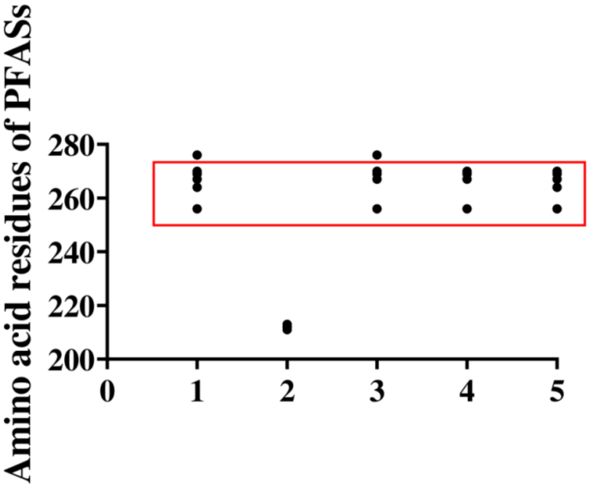

Figure S17. High-frequency amino acid distribution map of PFAAs in validation group：1. PFPA；2. PFOA；3. 5m-PFOA；4. PFOS；5. 6m-PFOS
